# Effect of Reference Genome Selection on the Performance of Computational Methods for Genome-Wide Protein-Protein Interaction Prediction

**DOI:** 10.1371/journal.pone.0042057

**Published:** 2012-07-26

**Authors:** Vijaykumar Yogesh Muley, Akash Ranjan

**Affiliations:** 1 Computational and Functional Genomics Group, Centre for DNA Fingerprinting and Diagnostics, Hyderabad, Andhra Pradesh, India; 2 Department of Biotechnology, Dr. Babasaheb Ambedkar Marathwada University, Sub-centre, Osmanabad, Maharashtra, India; The Centre for Research and Technology, Hellas, Greece

## Abstract

**Background:**

Recent progress in computational methods for predicting physical and functional protein-protein interactions has provided new insights into the complexity of biological processes. Most of these methods assume that functionally interacting proteins are likely to have a shared evolutionary history. This history can be traced out for the protein pairs of a query genome by correlating different evolutionary aspects of their homologs in multiple genomes known as the reference genomes. These methods include phylogenetic profiling, gene neighborhood and co-occurrence of the orthologous protein coding genes in the same cluster or operon. These are collectively known as genomic context methods. On the other hand a method called mirrortree is based on the similarity of phylogenetic trees between two interacting proteins. Comprehensive performance analyses of these methods have been frequently reported in literature. However, very few studies provide insight into the effect of reference genome selection on detection of meaningful protein interactions.

**Methods:**

We analyzed the performance of four methods and their variants to understand the effect of reference genome selection on prediction efficacy. We used six sets of reference genomes, sampled in accordance with phylogenetic diversity and relationship between organisms from 565 bacteria. We used *Escherichia coli* as a model organism and the gold standard datasets of interacting proteins reported in DIP, EcoCyc and KEGG databases to compare the performance of the prediction methods.

**Conclusions:**

Higher performance for predicting protein-protein interactions was achievable even with 100–150 bacterial genomes out of 565 genomes. Inclusion of archaeal genomes in the reference genome set improves performance. We find that in order to obtain a good performance, it is better to sample few genomes of related genera of prokaryotes from the large number of available genomes. Moreover, such a sampling allows for selecting 50–100 genomes for comparable accuracy of predictions when computational resources are limited.

## Introduction

In the last few years, computational methods of predicting physical and functional Protein-Protein Interaction (PPI) have gained popularity [1], [2], [3], [4], [5]. The interactions of an uncharacterized protein with known proteins in the predicted network often provide pointers for its functions [4], [6], [7], [8]. These networks also help in understanding the organization and the higher order functional relationships of proteins in various cellular processes [9], [10], [11], [12]. Most of these methods assume that functionally interacting proteins are likely to have a shared evolutionary history which can be traced out for all possible pairs of proteins present in the query genome (genome of interest). This is done by correlating different evolutionary aspects of their homologous proteins in multiple genomes referred to as reference genomes [7], [13], [14], [15], [16], [17]. These methods include phylogenetic profiling [14], [18], [19], [20], gene cluster [21], [22], [23], gene neighbor [24], [25], [26] and gene fusion [27]. They are collectively known as genomic context methods.

Phylogenetic profiling assumes that proteins gained or lost together during evolution are functionally interdependent and hence their co-occurrence is likely due to the mutual dependence. Phylogenetic profile or phyletic pattern is defined as a vector representing the presence or absence of a given protein in a set of reference genomes. Originally, the phylogenetic profile of a protein was represented qualitatively as a binary vector, where ‘1’ represented the presence of the protein in a reference genome and ‘0’ represented its absence [19]. Similarly, the presence of a given protein in phylogenetic profiles can also be quantitatively represented by transformed e-value scores and bit scores in the vector positions of the reference genomes [28], [29], [30]. The degree of similarity between phylogenetic profiles of two proteins reflects the strength of the functional association between them.

Chromosomal proximity of genes, irrespective of the relative gene orientation, has been shown to be an indicative of their co-regulation [26], as genes that participate in related biological processes are often observed to be co-regulated [31]. Hence chromosomal proximity of genes has been proposed as a parameter indicative of functional linkages. The genomic neighborhood of many prokaryotic genes have been broken down during evolution due to the frequently occurring dynamic re-arrangements [32], [33]. However, these rearrangements are conservative and maintain individual genes in very specific functional and regulatory contexts [24], [25]. Hence, it is possible to deduce these gene rearrangements based on chromosomal proximity of orthologous genes in multiple reference genomes. This approach is commonly referred to as the gene neighbor method [11], [25]. The gene cluster method also identifies the protein pairs that are encoded by neighboring genes on the reference genome sequence but they should be coded from the same genomic strand within a certain threshold of intergenic distance cutoff [21], [22], [23]. Therefore, this method discovers operonic rearrangements of a query genome based on the evidence of their operon structure in multiple reference genomes.

Another class of methods called mirrortree allows inference of physically interacting proteins based on the co-evolving amino acids in their protein sequences [34], [35], [36], [37], [38]. The assumption here is that the mutations in the residues responsible for interaction between two proteins may be compensated by complementary mutations to preserve or restore the interaction.

The performance of all these methods depends on the organisms selected for analysis, since the biological context of a protein is derived from the evolutionary information retained in the reference genomes. Therefore, we believe that the choice of reference genomes is one of the most critical parameters that can affect the performance of the aforementioned methods. However, most of the studies on the reference genome selection have been carried out for phylogenetic profiling [39], [40], [41], [42], [43], [44]. Jothi and coworkers analyzed the phylogenetic profiles constructed using a combination of 16 sets of reference genomes composed of eukaryotes, bacteria and archaea [40]. Their study suggested that the composition of the reference genome sets determines the prediction accuracy of the PPIs involved in various biological processes. Similarly, Anis-Karimpour and coworkers demonstrated the utility of phylogenetic profiles constructed from phenotypically and genotypically related organisms for prediction of PPIs that were missed when the reference genome set was assembled using phylogenetically diverse organisms [41]. A recent study on the genomic context methods also suggested a significant influence of the varying size and composition of the reference genomes on the prediction accuracy [45]. The mirrortree related methodologies were also tested for reference genome selection. It was observed that the certain subsets of reference genomes were more suitable for the predictions of certain types of interactions [39].

Our study focused on four methods that consider protein pairs and the evolutionary information of their orthologous pairs in various reference genomes to predict functional or physical linkages. Since their original implementations, these methods have diversified into a number of modified forms [22], [45], [46], [47], [48], [49]. We selected the variant methods that have not been evaluated against the effect of reference genome selection. These selected methods include variants of phylogenetic profiling, gene cluster and gene neighbor [22]. Apart from these genomic context methods, we have also studied mirrortree and Tree Of Life-mirrortree (Tol-mirrortree). We also introduced a new method to exclude speciation information called Genome Distance-mirrortree (GD-mirrortree) [34], [35]. We report comprehensive analyses of reference genome selection and its effect on prediction accuracy of the aforementioned prediction methods.

Considering the availability of a large number of completely sequenced genomes, it is challenging to select the organisms that would lead to the prediction of high-quality interactions. Furthermore, the processing time to compare these reference genomes is proportional to the number of genomes in the reference set. This study has important implications on the selection of reference organisms, a critical step in computational prediction of protein interactions.

## Results and Discussion

### Generation of reference genome sets

In order to evaluate the effect of reference genome selection on PPI predictions, the 565 reference genomes used in this study were grouped into six sets ALL, BAAC, BAS, BAC, GAMMA and BANR. The total number of genomes in “ALL” set included all the 565 prokaryotic genomes. Many genomes in ALL set were biased due to the presence of multiple species of the same genus. We created a “BAAC” set, which represented non-redundant 448 prokaryotic genomes, selected on the basis of shared *E. coli* orthologs. “BAS” set had a single genome of a particular genus and the closely related genera were removed. “BAC” set exclusively represented genomes of 86 phylogenetically diverse bacteria. Similarly, we created “GAMMA” set represented by 46 γ-proteobacterial genomes and “BANR” set represented by 41 reference genomes including 20 bacteria and 21 archaea. This filtering step was used to minimize the overrepresentation of certain genomes as many genera of prokaryotes have single species while others have multiple species. The composition in terms of phylogenetic distribution of each set is given in Table 1.

**Table 1:** Composition of reference genome sets used for analysis.

Class/Group	ALL	BAAC	BAS	BAC	GAMMA	BANR
Acidobacteria	2	2	0	1	0	1
Actinobacteria	46	37	9	7	0	2
Alphaproteobacteria	65	56	16	8	0	1
Aquificae	1	1	1	1	0	1
Bacteroidetes/Chlorobi	17	16	5	4	0	1
Betaproteobacteria	29	24	9	10	0	1
Chlamydiae	11	7	3	3	0	1
Chloroflexi	7	6	1	3	0	1
Cyanobacteria	29	24	7	4	0	1
Deinococcus-Thermus	3	2	1	1	0	0
Deltaproteobacteria	17	16	5	4	0	1
Epsilonproteobacteria	19	13	2	4	0	1
Firmicutes	128	96	17	9	0	4
Fusobacteria	1	1	1	1	0	1
Gammaproteobacteria	129	88	24	11	46	2
Other Bacteria	3	3	0	3	0	2
Planctomycetes	1	1	1	1	0	1
Spirochaetes	5	4	2	5	0	1
Thermotogae	6	5	1	6	0	1
Nanoarchaeota*	1	1	1	0	0	1
Crenarchaeota*	15	15	3	0	0	3
Euryarchaeota*	30	30	13	0	0	13
Total number of reference genomes	565	448	122	86	46	41
Total number of protein sequences (in thousands)	1734	1389	364	265	151	113

Notes: ALL - All prokaryotic genomes; BAAC – Automatically selected diverse prokaryotic genomes (see method for details); BAS – Only single representative genomes of species from same genus and related genera; BAC – Non-redundant bacterial genomes; GAMMA – Non-redundant γ-proteobacterial genomes; BANR – Non-redundant Bacterial and Archaeal genomes. Asterisk marks represent groups that belong to Archaea super-kingdom. Classification is extracted from http://www.ncbi.nlm.nih.gov/genomes/lproks.cgi.

### Gold standard dataset used for comparisons

In order to evaluate the effect of reference genome selection on the accuracies of PPI prediction methods, we required gold standard datasets. Two gold standard datasets were created using Database of Interacting Partners (DIP), EcoCyc protein complexes and Kyoto Encyclopedia of Genes and Genomes (KEGG) pathway annotations [50], [51], [52]. Our first gold standard dataset, called High-Quality Gold standard (HQG), consisted of positive protein pairs (for which the orthologs were present in 200 or more genomes) with the evidence of physical and/or complex associated interactions and they belonged to the same functional category according to EcoCyc or KEGG pathway annotations. Our second gold standard dataset called Low-Quality Gold standard (LQG), was a union of the above three resources without phyletic distribution constraint as mentioned above. The phylogenetic distribution constraint for HQG was applied in order to ensure that only genomic signals and not the phyletic distribution of proteins determine the prediction accuracy. For obtaining a negative dataset, the simplest way is to generate all possible pairs among the proteins of an organism and then remove all potential positive pairs from that dataset. The remaining pairs can then be used as negative datasets given that the partners in each pair should neither be present in the same pathway nor in the same subcellular compartment [53]. This additional filtering step is recommended due to incomplete knowledge of actual positive dataset. We first generated all possible protein pairs among the proteins that constituted positive pairs for HQG dataset and then removed positive pairs from the same. The resulting subset was used as negative examples for HQG dataset. We also cross-checked results obtained on HQG and LQG datasets with the complete set of DIP and EcoCyc co-complex PPIs (referred hereafter EcoCyc) independently.

### Effect on Phylogenetic Profile Method (PPM)

Previous studies have used the transformed e-values to create phylogenetic profiles since the authors in such instances believed that e-value measures sequence divergence [30], [40], [54], [55]. We suggest that the e-value based phylogenetic profiling does not capture sequence divergence information as well as bit score profiling does. E-value is a measure of the probability that a given BLAST search hit is obtained by chance for a size of a given database whereas bit score is a normalized sequence similarity score representing the quality of the match based on sequence alignment [56]. Hence in our opinion, it is preferable to construct phylogenetic profiles using bit scores as opposed to transformed e-values to capture sequence divergence in a better way. The results obtained using bit score profiles were compared with that of binary profiles since the previous studies lack such comparisons with respect to the reference genome selection. In our study, the interaction scoring using binary profiles is referred to as Binary Phylogenetic Profile Method (BPPM) whereas interaction scoring using normalized bit scores is referred as Sequence similarity based Phylogenetic Profile Method (SPPM).

#### SPPM outperforms BPPM for all reference genome sets

**Figure 1:**
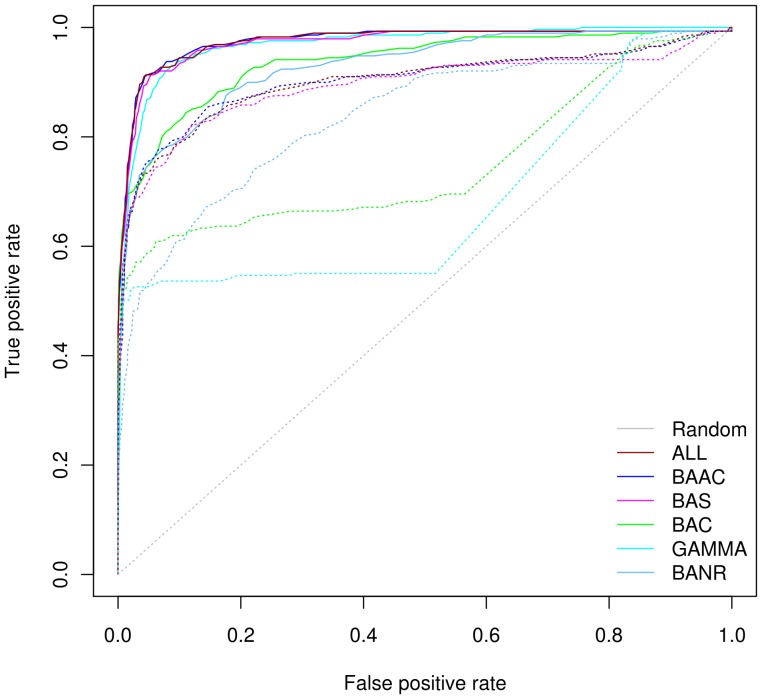
ROC curves for six reference genome sets using Phylogenetic Profiling Methods. The solid lines depict the phylogenetic profile constructed using normalized bit scores (SPPM) whereas the dotted lines depict the binary phylogenetic profile (BPPM). The colors of the lines correspond to the six reference genome sets (ALL, BAAC, BAS, BAC, GAMMA and BANR) for which performance was evaluated. As evident in the figure, SPPM gives superior performance compared to BPPM for all reference genome sets. The ROC curves clearly show that the reference genome selection has profound influence on the performance of BPPM compared to that of SPPM.

We have compared the prediction accuracy of BPPM and SPPM using Receiver Operating Characteristic (ROC) curves. SPPM outperformed BPPM for all the six reference genome sets that we tested (Figure 1). The performance of SPPM was almost similar for ALL, BAAC, BAS and GAMMA reference genome sets. All these sets achieved AUC value of 0.97 (Table 2). The performance of BAC (AUC 0.94) and BANR (AUC 0.93) was poorer than that of the above mentioned sets. The poor performance of BANR was expected due to its small size (41 genomes) and the inclusion of almost equal proportion of bacterial and archaeal genomes. However, the poor performance of BAC with respect to that of GAMMA was intriguing. BAC set represented diverse bacterial genomes whereas GAMMA was formed by an exclusive set of γ-proteobacteria to which the query organism *E. coli* belongs. Upon close inspection of the phyletic distribution of the numbers of genomes in each set, it was observed that the BAC set included only 11 γ-proteobacterial genomes as compared to the 129, 88, 24 and 46 genomes in ALL, BAAC, BAS and GAMMA sets respectively (Table 1). We corroborated these numbers with the AUC values (Table 2) which suggests that an inclusion of higher number of γ-proteobacterial genomes in various reference sets improved the performance of the SPPM. It has been observed that phylogenetic profiling using a diverse set of genomes gives better performance accuracy [40], [55], [57]. However, our results suggest that the closely related genera are providing evolutionary information resulting in a better performance in the case of SPPM. Our results were consistent with an observation of a previous study [41] that many unique interactions were obtained when closely related genomes constituted the reference set. While these interactions were missed if distantly related genomes were used. Even with EcoCyc gold standard we observed that AUC value for GAMMA (0.87) was better than that of BAC (0.83) set (Table S3). However, in the case of DIP we observed AUC values were comparable (Table S2). Overall, these results reflect relatively similar performance of SPPM irrespective of the size and the composition of the reference genome sets.

**Table 2:** Performance summary for four computational methods and their variants using six reference genome sets.

Method	Variant	ALL	BAAC	BAS	BAC	GAMMA	BANR
PPM	BPPM	0.90	0.90	0.89	0.75	0.68	0.84
	SPPM	0.97	0.97	0.97	0.94	0.97	0.93
GCM	GCM	0.76	0.76	0.78	0.76	0.80	0.75
MDM	MDM	0.88	0.88	0.90	0.89	0.90	0.90
Mirrortree	Mirrortree	NA	NA	0.90	0.90	0.78	0.84
	Tol-mirrortree	NA	NA	0.82	0.91	0.80	0.74
	GD-mirrortree	NA	NA	0.94	0.92	0.86	0.81

Notes: The performance summary of protein-protein prediction methods measured as Area Under the ROC Curve (AUC). BPPM stands for Binary Phylogenetic Profile Method; SPPM stands for Sequence Similarity (bit scores) based Phylogenetic Profiling Method; GCM is Gene Cluster Method, MDM is gene neighborhood based Minimum Distance Method; GD is genome distance; NA stands for sets that are not analyzed for corresponding method. ALL, BAAC, BAS, BAC, GAMMA and BANR are reference genome sets whose compositions is given in Table 1.

#### Performance of BPPM is influenced by reference genome set

ROC curves of BPPM show almost similar performance for ALL, BAAC and BAS sets (Figure 1). The AUC value for ALL and BAAC was 0.90 whereas for BAS was 0.89 (Table 2). The relative trends of ROC curves for BPPM using BAC, GAMMA and BANR (to some extent) sets showed a wide variation compared to SPPM curves. It reflects the influence of reference genome selection on the BPPM performance. BANR achieved AUC value of 0.84 whereas BAC and GAMMA sets showed worse performance with AUC values of 0.75 and 0.68 respectively. The TPR values of BAC and GAMMA sharply decline when the BPPM interaction scores are relaxed. The number of genomes in BAC and GAMMA sets was less compared to that of ALL, BAAC and BAS (Table 1). The effect of higher number of genomes on the performance of PPM has been controversial as some reports have suggested a better prediction accuracy is associated with higher number of genomes in the reference set [44], [55] while Jothi and coworkers have contradicted it [40]. Our results are in agreement with the former observation when HQG and EcoCyc datasets were used (Table 2 & Table S3). Counter-intuitively, BPPM performance using a small number of genomes was comparable with higher number of genomes, provided the set included phylogenetically distant genomes. This is due to the fact that BANR (41 genomes) set which was composed of an almost equal proportion of bacterial and archaeal genomes predicted interactions with the AUC value of 0.84 which was less but comparable to the same achieved for ALL (565 genomes), BAAC (448 genomes) and BAS (122 genomes) (Figure 1). So the contradictory observation made by Jothi and coworkers is possibly due to the selection of phylogenetically diverse sets of 95 organisms which consists of representative organisms from various branches of the canonical tree of life in their study [40]. This can be supported by the fact that performances of BAC (86 genomes) and GAMMA (46 genomes) were poor as compared to BANR (41 genomes). These observations reveal the fact that close relatives of query genome are not suitable as reference organisms for binary phylogenetic profiling and would probably result in the over scoring for functionally unrelated protein pairs [57]. We made similar observations for BAC and GAMMA sets using LQG and DIP while EcoCyc showed a comparable AUC score. Overall, our results suggest that performance of BPPM is profoundly dependent on the reference genome selection as compared to the SPPM. It is mostly influenced by inclusion of closely related genomes. However, it was unclear whether small size of reference genome selected from distantly related species or the set with higher number of genomes is responsible for higher performance accuracy of BPPM, since different gold standard datasets were supporting both the findings (Table 2,S1,S2 & S3).

#### Bit scores used in SPPM reduce the influence of reference genome selection

As explained above, the performance of the BPPM was influenced by the composition and the size of reference genome sets. The BPPM AUC values for BAC and GAMMA sets were 0.75 and 0.68 respectively. The reason for the poor performance of BAC and GAMMA reference sets in case of BPPM was possibly due to the profiles containing runs of ‘1’ for several proteins of *E. coli* which were shared among bacterial and the sub-class γ-proteobacterial lineage respectively. Although these sets include only the representative species but still due to close relatedness to *E. coli*, these species share many common genes and hence result into similar phylogenetic profiles for a number of proteins irrespective of their functional relevance [40], [57]. It might be a case for the worst performance of GAMMA when BPPM was used for prediction.

Compared to BPPM, SPPM was robust against reference genome selection with comparable performance accuracy for each reference genome set (Figure 1 and Table 2, S1,S2,S3). We speculate that the bit score based profiles minimize the effect of reference genome selection. Furthermore, we performed two normalizations on the phylogenetic profile matrix [22], [29]. First normalization was performed for a particular *E. coli* protein profile using the maximum bit score obtained over all its orthologs in a given reference genome set. The second normalization was performed using the minimum bit score which was obtained from all the orthologs in a particular genome in a given reference set. These two steps minimized the effect of higher protein sequence divergence and species divergence. Therefore the phylogenetic profiles constructed using bit scores followed by double normalization are expected to contain information of amino acid changes due to functional constraints instead of speciation events. Our results supported the argument put forward by Kensche and coworkers that the bit score representation of phylogenetic profiles gets the benefit of sequence similarity in addition to co-occurrence of proteins [20] and thereby further improves the information content [22], [29]. Similar results were obtained using the LQG dataset as shown in Figure S1A and Table S1. PR curves also led to the same results as observed for ROC measures (Figure S2A). Our results remained broadly consistent when cross-checked on DIP and EcoCyc. We observed that average AUC values remarkably differ for BPPM when BAC and GAMMA sets were used as reference, as compared to that of SPPM for the same sets (Table 2,S1,S2 & S3).

### Effect on Gene Cluster Method (GCM)

Gene cluster is defined as a set of consecutive co-directional genes with intergenic distance(s) less than a certain threshold nucleotide bases in a microbial genome sequence [21], [58]. For given two proteins, GCM calculates the probability of co-occurrence of genes encoding their orthologs in the same gene clusters in the reference genomes [22]. Thus, GCM identifies operons rearranged in a query genome during evolution. In our analysis, gene clusters were defined in all reference genome sets using intergenic distance threshold of 100 nucleotide bases and propensity scores were calculated for all gold standard protein pairs. As the intergenic distance threshold of 100 nucleotide bases gave accuracy better than that of 200, 300, 400 and 500 (Table S4).

#### GCM is a highly specific predictor of functionally linked proteins

It was observed that the propensity scores calculated for the positive examples of the gold standard datasets were very low. Surprisingly, we observed few negative examples with the propensity scores above zero which were not enough to evaluate GCM performance (Table 3). It also suggested that negative examples chosen for evaluation were likely to be non-interacting. The higher propensity scores for *E. coli* protein pairs reflect the frequent co-occurrence of their ortholog encoding genes in the same cluster. It suggests their coupled transcription in reference genomes. Therefore, the higher propensity scores for positives than that of negative examples (mostly with score zero) suggesting the reliability of negative pairs in the gold standard dataset and the higher specificity of GCM.

**Table 3:** Gold Standard protein pairs with GCM propensity scores above zero for six reference genome sets.

Dataset	ALL	BAAC	BAS	BAC	GAMMA	BANR
LQG	2312/363	2306/357	1948/182	1834/172	1606/81	1403/67
KEGG	4507/15396	4465/15114	3299/7338	2942/6019	2152/2574	2036/2873

Notes: ALL, BAAC, BAS, BAC, GAMMA and BANR are reference genome sets whose compositions is given in Table 1. Numbers represent positive/negative pairs with GCM scores above zero for KEGG and LQG datasets.

In order to address the problem of less number of negative examples for evaluation, pathway similarity scores for 1,013,176 possible pairs among 1,424 proteins annotated in KEGG database were calculated using Jaccard coefficient [30]. The protein pairs having the pathway similarity scores and the GCM scores above zero were treated as positive examples. While the protein pairs having the pathway similarity scores equal to zero and the GCM scores above zero were treated as negative examples. These pairs used for evaluation and were termed as the KEGG gold standard dataset (Table 3).

#### GCM performance is better using closely related species in reference genome set

**Figure 2:**
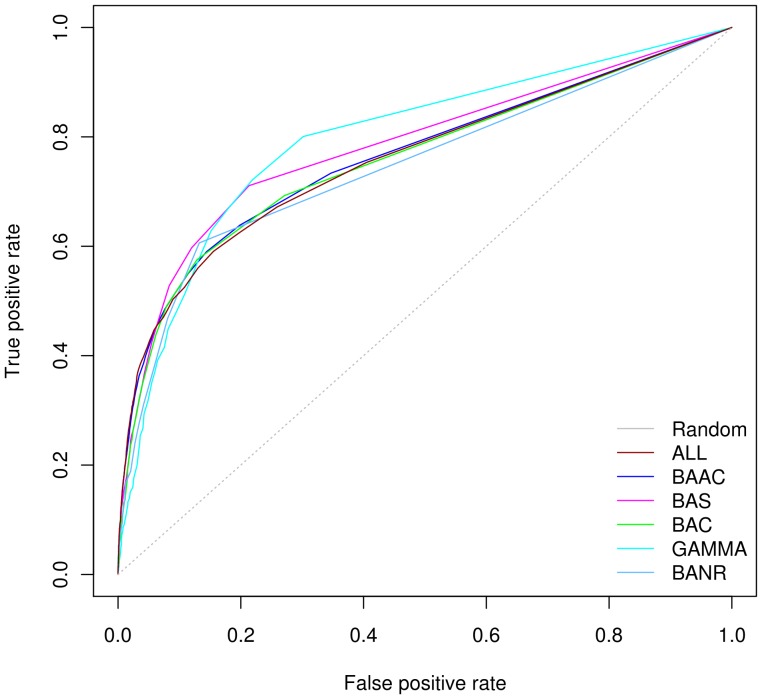
ROC curves for six reference genome sets using Gene Cluster Method. The colors of the lines correspond to the six reference genome sets (ALL, BAAC, BAS, BAC, GAMMA and BANR) for which performance was evaluated. The reference genome set GAMMA relatively performs better.

The performance of GCM in the low FPR region was better for the sets that consisted of higher numbers of genomes (Figure 2). For full range, GAMMA and BAS sets outperformed the other sets. AUC values, for six reference genome sets, ranged from 0.75 to 0.80 (Table 2). The highest AUC value was obtained for GAMMA set whereas the lowest was obtained for BANR. The better performance of GAMMA could be explained by the fact that the gene order, gene content and regulatory mechanisms of operons are not conserved even in closely related species [23], [24], [32], [59]. In other words, reference sets containing closely related genomes provide many gene clusters or operons that are rearranged in the distantly related ones. Furthermore, the number of positives (2152 pairs) of KEGG gold standard compared to the negatives (2574 pairs) with GCM score above zero was better when GAMMA set was used (Table 3). This proportion of positives and negatives achieved by GCM is much better than the BANR set with 2036 positives and 2873 negatives. The BANR set include 41 most distantly related genomes. However, this observation is consistent only with evaluation on DIP, while performance for ALL reference genome set was better when EcoCyc used as benchmark (Table S2 & S3).

ROC curves show similar trends for four out of six reference genome sets (Figure 2). The difference became more apparent when PR was used as the complementary performance measure and HQG as benchmark. PR curves suggest that performance of all the six sets in the region of high precision values and less recall (i.e. the region where GCM scores are high) is almost similar (Figure S2B). We observed recall of 0.20 at 0.90 precision in this region reflecting very few falsely predicted interactions. Remarkably, the PR curves reached 100% recall for majority of the reference genome sets well above the precision value of 0.3. From these observations, we suggest that reliable predictions could be achieved using GCM that are likely to be free of false positives.

Overall, the outperformance of GAMMA reference set of closely related genomes to the query genome was unexpected. However, KEGG and DIP gold standards support these findings. Therefore, the highest accuracy for GAMMA set could be attributed to the higher numbers of intact neighborhoods with capacity to encode functionally related proteins in closely related genomes as compared to the distantly related ones.

### Effect on Minimum Distance Method (MDM)

**Figure 3:**
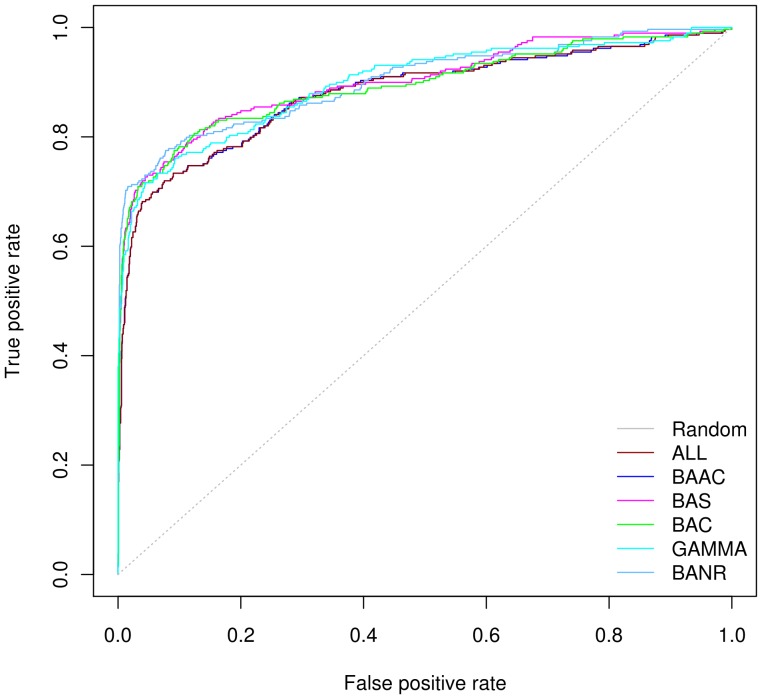
ROC curves for six reference genome sets using Minimum Distance Method. The colors of the lines correspond to the six reference genome sets (ALL, BAAC, BAS, BAC, GAMMA and BANR) for which performance was evaluated. ROC plot shows that the method is robust against choice of reference genome sets. All reference sets performed equally well.

MDM method identifies proteins that are no longer encoded by neighboring genes in the query genome but genes encoding their orthologs are present in proximity in any one genome of the reference set [11], [22]. ROC curves for MDM suggest substantial similarity in the performance of each reference genome set (Figure 3). The range of AUC values obtained fall in between 0.88 to 0.90 (Table 2). These results suggest that the performance of MDM is not influenced by reference genome selection. MDM calculates an interaction score based on the minimum chromosomal distance between two genes from any one genome probably making MDM less sensitive to the reference genome set. The performance of MDM on LQG dataset also showed robustness against the reference genome selection (Figure S1B). At 0.20 FPR we found that BAS set performed reasonably well on HQG and LQG with TPR of 0.86 and 0.53, respectively.

MDM is a variant of Gene Neighbor Method (GNM) and the previous report suggested that the GNM outperforms PPM [45]. Contrary to the previous report, we observed that the performance of PPM is substantially better than MDM when HQG dataset was used for evaluation (Table 2). On the LQG dataset, however, we observed that the performance of MDM was slightly raised over PPM and which is consistent with reference [45]. We confirmed that this difference was due to the gold standard dataset used for evaluation. Unlike the HQG dataset which consisted of physical PPIs, the LQG positives were dominated by KEGG pathway PPIs i.e. out of 7,217 positives, 6240 were KEGG pathway pairs. Similarly, Ferrer and coworkers used gold standard set, which was mostly composed of known enzymes that participates in various metabolic pathways [45]. The effectiveness of GNM to predict metabolic PPIs is observed in previous studies [25], [11]. Recent analysis suggested GNM was the most effective method to reconstruct metabolic pathways based on chromosomal proximity of proteins [48]. Therefore, the outperformance of MDM/GNM over PPM on the LQG or the gold standard used in the previous study is not surprising [45]. Gene neighbor variants would always outperform PPM when it comes to predictions of metabolic PPIs due to the independent evolutionary histories of metabolic pathways [40].

We observed more or less similar AUC values for six reference genome sets when cross-checked with DIP and EcoCyc (Table S2 & S3).Therefore the reference set with 50–150 phylogenetically diverse prokaryotic genomes would be a good choice for high confidence predictions using MDM. PR curves also led to the same results as observed for ROC measures (Figure S2C).

### Effect on Mirrortree Based Methods

The mirrortree method compares the similarity between two sets of distance matrices computed for potentially interacting protein pair using a correlation coefficient, which is indicative of similarity in phylogenetic trees and hence suggesting a possible co-evolution. These distance matrices were computed from Multiple Sequence Alignment (MSA) for each protein pair of query organism (*E. coli*) [34], [60]. The matrix for each protein represented distances among amino acid sequences of its orthologs. Being mindful of the computational complexity and time requirement for MSAs construction, we carried out an analysis for 122 reference genomes represented in the BAS set. The BAS set was further sub-divided into four reference genome sets based on phylogenetic diversity (Table 4).

**Table 4:** A statistical summary of reference genome sets used for mirrortree based analyses.

Reference genome set	Number of genomes		Scaling factor	
	GD-mirrortree	Tol-mirrortree	GD-mirrortree	Tol-mirrortree
**GAMMA**	24	24	0.50	0.66
**BANR**	36	34	0.37	0.38
**BAC**	66	66	0.45	0.47
**BAS**	122	120	0.54	0.89

Notes: BAS, BAC, GAMMA and BANR are reference genome sets. GD is genome distance. Scaling factor is the highest correlation coefficient obtained between GD or 16S rRNA distance matrix when compared with protein distance matrices derived for each reference genome set. Tol-mirrortree analysis using BANR and BAS set performed using 34 and 120 reference genomes since 16S rRNA sequences for two Archaeal genomes could not be retrieve from Ribosomal database [69].

In order to exclude the background similarity due to the underlying speciation events, one can correct the protein distance matrices using various approaches [35], [61]. It is suggested that a 16S rRNA based correction of distance matrices of protein families represents a coordinated evolutionary history and does not contain speciation information. This approach improves the prediction accuracy and it is called as Tol-mirrortree method [35]. However, we think that the organisms thriving in a specific ecological niche evolve traits, which can help them withstand the surrounding environmental conditions [62]. Such events in the evolutionary history of organisms are taking place at protein or gene level and not at the non-coding 16S rRNA sequence level. Hence this information can be better captured by comparing total protein content of reference genomes with one another. We suggested an alternate correction approach based on comparison of proteins present in the reference genomes termed as GD-mirrortree.

#### GD-Mirrortree outperformed Tol-mirrortree

**Figure 4:**
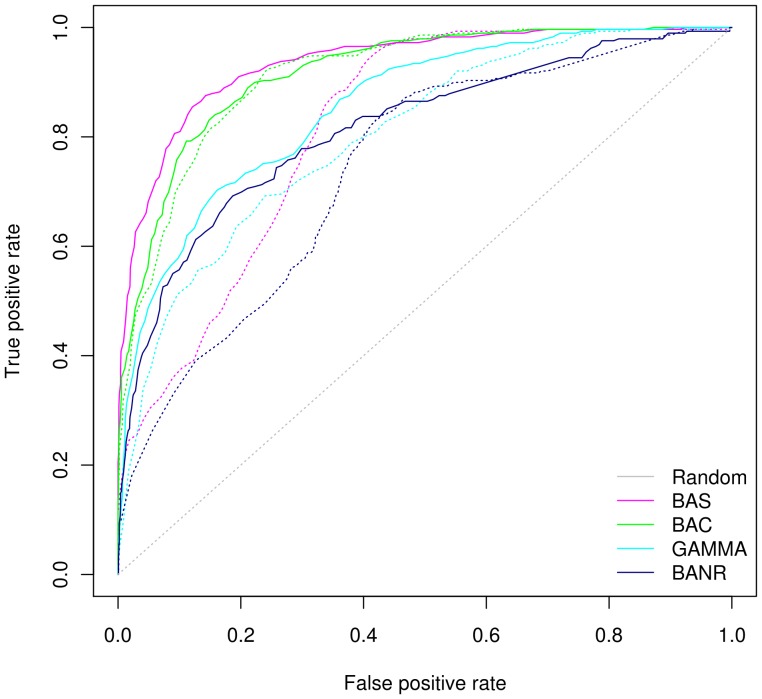
ROC curves for four reference genome sets using Mirrortree based methods. We have used here two variants of the mirrortree methods i.e. the Tol-mirrortree and GD-mirrortree. The Tol-mirrortree (represented by dotted lines in the plot) uses 16S rRNA distance between two genomes as a factor to correct the phylogenetic distance whereas the GD-mirrortree (represented by solid lines in the plot) uses a genomic distance parameter reflecting the shared orthologs between two genomes to correct the corresponding phylogenetic distance (See methods for detail). The colors of the lines correspond to the four reference genome sets (BAS, BAC, GAMMA and BANR) for which performance was evaluated. The plot clearly shows that the GD-mirrortree method is superior to Tol-mirrortree method for these four reference genome sets. BAS and BAC perform better than GAMMA and BANR with comparable level of accuracy.

ROC curves shown in Figure 4 illustrate the superiority of GD-mirrortree method compared to Tol-mirrortree. Our GD-mirrortree approach outperformed Tol-mirrortree using all reference genome sets. The best performing reference genome set in case of GD-mirrortree was BAS whereas for Tol-mirrortree was BAC. ROC curves of BAC set for both approaches were almost similar and to some extent to that of GAMMA set. However, as the interaction scores calculated by Tol-mirrortree were relaxed, there was a decline in the TPR for BAS and BANR reference genome sets whereas curves of BAC and GAMMA sets were relatively stable and gradually increased. We observed that the corrections using 16S rRNA distances performed efficiently only when the phylogenetic distances among the organisms in the reference set were low. ROC curves of GD-mirrortree method for various reference genome sets were quite stable for full range of calculated interaction scores as compared to the behavior of Tol-mirrortree (Figure 4). The prediction accuracy was observed highest for BAS and lowest for BANR suggesting that the higher number of genomes in the reference set was better for prediction accuracy. PR curves also led to the same results as observed for ROC measures (Figure S2D).

Performance results are reported in the form of AUCs for mirrortree, Tol-mirrortree and GD-mirrortree in Table 2. It was expected that performance of Tol-mirrortree would be better than mirrortree [35]. Our results suggest that the prediction accuracy of Tol-mirrortree was better than mirrortree but not for all reference genome sets. Tol-mirrortree performed better than the mirrortree for BAC and GAMMA sets. However, Tol-mirrortree showed poor performance than the mirrortree for BAS and BANR sets with AUC values of 0.82 and 0.74 compared to 0.90 and 0.84 respectively. It suggests that the bias caused by closely related genomes was effectively corrected by Tol-mirrortree. However, if the reference genome set contained distantly related genomes (as in case of BAS and BANR), then the 16S rRNA distance based correction caused deterioration of performance.

The aforementioned results remain broadly consistent even when DIP and EcoCyc gold standard datasets were used (Table S2 & S3). However, on LQG dataset the performance of GD-mirrortree and Tol-mirrortree methods was better for BAC and BANR than that of BAS and GAMMA (Figure S1C). Nonetheless, GD-mirrortree again performed slightly better than Tol-mirrortree. The probable reason for such a discrepancy of ROC curves on LQG dataset is unexplainable. We believe that the source of such discrepancy was 80% positive pairs of LQG that consist of functional interactions of proteins co-occuring in the same KEGG pathways, whereas inherently, mirrortree based methods are known to be predictors of physical interactions.

### Conclusions

The optimal performance of phylogenetic profiling, gene neighbor, gene cluster and mirrortree methods for protein-protein interaction predictions depends on the evolutionary information retained in reference genomes selected for analysis. We compared performance of these methods and their variants using carefully chosen six reference genome sets in accordance with phylogenetic diversity and show that all methods except GCM showed substantially improved performance when a subset of phylogenetically diverse archaeal genomes was used with eubacteria. Phylogenetic profiling using bit scores as compared to the binary digits performed relatively similar for all reference genome sets. We conclude that the use of sequence similarity scores (bit scores) to construct phylogenetic profiles minimizes the effect of reference genome selection. Likewise, the gene neighbor variant used in our study also showed robustness against the reference genome selection. Arguably, our study suggests that the gene cluster method performs best using reference set of genomes that are phylogenetically close relatives of the query organism.

We have verified these results on number of gold standards and found comparable results. There were subtle differences in the performance of various methods when different gold standards were used for evaluation of reference genome sets. Therefore, we presented results that were derived from majority of gold standard datasets. Among other sets, the BAS reference set of 121 phylogenetically diverse genomes gave accuracy comparable to that achieved when other reference sets with about four times higher numbers of genomes were used. Hence, it can be inferred that the set with 100–150 genomes from each genus and related genera representing all known classes/groups of prokaryotes should be good enough to predict interactions with high accuracy. Notably, the variants of phylogenetic profiling, gene neighbor and gene cluster methods analyzed in our study can be used effectively for protein-protein interaction predictions with small subset of available several hundred prokaryotic genomes. In fact, phylogenetic profiling and gene neighbor variants should work with any combination of reference genomes. Our observations are limited to eubacterial query genome and prokaryotic genomes as the reference set. Therefore, it would be interesting to study whether these observations also hold true for other two domains of life i.e. eukaryotes, and archaea.

## Materials and Methods

We chose the genome of *Escherichia coli* K12 MG1655 (*E. coli*) as the query genome. Completely sequenced genomes of bacteria available as on December 2007 at National Center for Biotechnology Information (NCBI) were downloaded from ftp://ftp.ncbi.nih.gov/genomes/Bacteria [63]. A total of 566 prokaryotic species with single chromosome were used for analysis. Orthologs of each *E. coli* protein were identified by performing reciprocal best hit search using standalone BLAST against the remaining 565 genomes [56]. The reciprocal hits with e-value less than or equal to 1e-4 were retained as potential orthologs of *E. coli* proteins.

Given two proteins X and Y of the query genome, each prediction method generates a numerical value based on various aspects of their evolution computed through orthologs in a set of reference genomes referred as interaction score. The interaction score reflects the degree with which two proteins are functionally linked. Considering the computational resources required to compute the interaction scores for all possible pairs of *E. coli* proteins, we performed analyses only for protein pairs that are identified as positive and negative gold standard (explained in next section).

Each prediction method requires a set of reference genomes to compute interaction scores. We created six sets of reference genomes from initial set of 565 that are called ALL, BAAC, BAS, BAC, GAMMA and BANR (Table 1). BAAC set is composed of 448 diverse reference genomes, automatically detected based on the shared orthologs of *E. coli* proteins between them. To remove reference genomes with similar proportion of orthologs detected in *E. coli*, a fraction of similarity between the two reference genomes was calculated using Tanimoto coefficient as follows,


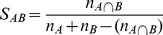


Where *n_A_* and *n_B_* are the number of *E. coli* orthologs in reference genomes A and B respectively. *n*_A∩B_ is the number of *E. coli* orthologs shared by genomes A and B [64]. The resulting Tanimoto coefficients (*S_AB_*) between all possible pairs of reference genomes were sorted and those having coefficient of 0.9 or more were selected for clustering. The clustering was carried out using Markov Cluster algorithm (MCL) [65] and only one genome was retained from each cluster. A total of 448 genomes remained after filtering 117 out of 565 original reference genomes.

### Gold standard dataset for evaluation of prediction methods

Physical and functional interactions reported in the EcoCyc database (version 13), the DIP (January 2009 version) and the KEGG database were used to create a gold standard dataset [50], [52], [66]. First, we extracted 1,072 and 55,779 pairs constituting proteins that co-occur in the same EcoCyc protein complexes or KEGG pathways, respectively, 541 physically interacting protein pairs reported in DIP with evidence of low throughput experimental analysis, and 3,873 functionally associated protein pairs from EcoCyc database. Using these proteins pairs, we constructed two positive (i.e. proteins that interact) gold standard datasets. First set consisted of 289 pairs that are a part of DIP or protein complex and have been reported in either KEGG or EcoCyc functional interactions. So each protein pair of this dataset had evidence of their physical association and participates in the same functional pathway. Prior to selection, proteins with their orthologs in less than 200 reference genomes were removed. We referred this dataset as High Quality Gold standard (HQG) dataset. Second set of 7,217 positive examples were created by combining interactions reported in DIP, Complex and KEGG dataset. As stated above, KEGG constitutes 55,779 protein pairs whose proteins co-occur in at least one KEGG pathway. It is possible that one protein can participate in multiple pathways. Hence, we only considered 6,240 protein pairs that participate in only one KEGG pathway. This dataset was referred as Low-Quality Gold standard dataset (LQG).

For testing the prediction methods, it was necessary to have protein pairs that do not interact with each other, i.e., negative examples. Defining reliable negative examples for predicting PPI has been acknowledged to be a challenging task [53], [67]. As described in previous studies, the negative dataset was formulated based on different sub-cellular localization [22], [53]. The presence of signal sequence and transmembrane helix were predicted in all *E. coli* proteins using Phobius web server (http://phobius.sbc.su.se/) [68]. There is always a possibility of false prediction of signal sequences as a transmembrane helix or vice-versa due to the presence of hydrophobic amino acids in their sequences. Therefore, the proteins with the presence of both signal sequence and transmembrane helix or only transmembrane helix were removed. Proteins that belong to more than one KEGG third level categories were also removed to avoid possible functional overlap [53]. Remaining proteins from different KEGG III level categories were paired to form 3,52,673 potential non-interacting protein pairs, each protein in a pair was from a different sub-cellular localization (e.g. secretory and cytosolic).

We randomly selected 1,445 and 36,085 negative examples among the proteins that constituted HQG and LQG positive examples respectively. These negative pairs were incorporated into HQG and LQG dataset to make the ratio of positive versus negative examples 1∶5 which resulted into 17,34 and 43,302 protein pairs in HQG and LQG dataset for evaluation respectively. Additionally, we used complete DIP and EcoCyc co-complex PPIs as gold standards to cross-validate the results obtained by HQG and LQG datasets. The positive datasets then combined with 1,14,504 negative protein pairs that belong to the different sub-cellular localization and the different functional categories at the first level of KEGG Orthology definition [50].

### Computation of interaction scores

Interaction scores for gold standard protein pairs were calculated using variants of five prediction methods which include phylogenetic profiling, gene cluster, gene neighbor and mirrortree as follow,

#### Phylogenetic Profiling Method

Phylogenetic profile matrices were created for six reference genome sets. Rows in such matrices were *E. coli* proteins, *i_1_, i_2_…i_4132_* and columns were reference genomes, *j_1_, j_2_…j_n_*, where *n* is the number of genomes in a reference set. Each (*i,j*) cell of this matrix was filled with the bit score of *E. coli* protein *i* and its homolog in the *j* ^th^ reference genome. If a protein was absent in any reference genome then it was denoted with score zero. Each cell or point of the phylogenetic profile matrix of a protein, *i* (i.e., row) was normalized as NBS*_ij_* = BS*_ij_*/BS*_max_*, where BS*_ij_* is the bit score of the alignment between *E. coli* protein *i* and its ortholog in reference genome *j*. BS*_max_* is the maximum value of bit score obtained for protein *i* over all its orthologs from *n* reference genomes. Second normalization was carried out on reference genomes (i.e., column) by dividing the minimum bit score over all *E. coli* protein orthologs in *j* ^th^ reference genome [22], [29]. Likewise, another set of profile matrices created as a control where the presence and absence of *E. coli* protein orthologs in matrix was represented with ‘1’ and ‘0’, respectively [19]. Two proteins X and Y of *E. coli* displaying similar phylogenetic profiles were assessed by calculating standard Pearson Correlation Coefficient (PCC) between their vectors.

#### Gene Cluster Method (GCM)

A gene cluster in a genome is defined as a set of continuous co-directional genes with an intergenic distance of 100 nucleotide bases or less between them. The gene clusters were identified in all the reference genomes. The propensity scores for gold standard protein pairs were calculated as,


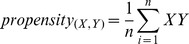


Where *n* is the number of genomes in a reference set, XY = 1 if orthologs of *E. coli* protein X,Y∈gene cluster in *i* ^th^ reference genome, otherwise 0 [22].

#### Minimum Distance Method (MDM)

The minimum distance between genes encoding protein X and Y of *E. coli* on the basis of genes encoding their orthologs in the reference genomes is calculated as described in [22]. Briefly, if the query proteins X and Y are present in the reference genome *i*, then the probability that genes encoding their orthologs are separated by fewer than *d* nucleotide bases is given by


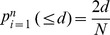


Where, *d* is the distance between translation start sites of genes encoding orthologs of X and Y in the *i* ^th^ reference genome. *N* is the length of the chromosome of *i* ^th^ reference genome in nucleotide bases. *n* is the total number of reference genomes in a set. Since the genomes under consideration are circular, the distances between the gene pairs were calculated in both clockwise and anti-clockwise direction. Minimum of these two values is *d*. The minimum probability in any one reference genome is considered as the interaction score for query proteins X and Y.

#### Mirrortree based methods

To quantify the co-evolution of amino acids, Multiple Sequence Alignments (MSA) of the proteins with their orthologs obtained from reference genomes were generated. Then the distance matrices derived from the MSAs were compared to find out the extent of co-evolving amino acids. Considering the high computational cost of MSA construction, *E. coli* proteins and their orthologs selected from 122 reference genomes only (i.e., BAS set) were used for the construction of MSAs by ClustalW [69]. Phylogenetic distance matrices were generated for each *E. coli* protein using their MSAs. Each protein matrix was of size *n*×*n*, where *n* represents the number of reference genomes in which orthologs were detected. An element of the distance matrix *D* for protein X, i.e. *D*X(*i,j*), represented the genetic distance between reference genomes *i* and *j*, which is a difference in amino acid sequences of protein X from reference genome *i* and *j*.

Distance matrices of two proteins, X and Y are only comparable when their dimensions are same. However, dimension of each protein matrix may differ depending on its phyletic distribution in the reference genome set. Similar to the original implementation of mirrortree approach, we considered a minimum of 15 common reference genomes between distance matrices of both proteins to calculate PCC between them [34]. We applied Tol-mirrortree and a variant of this method referred here as GD-mirrortree to exclude speciation information from these distance matrices.

In Tol-mirrortree method, protein distance matrices were rescaled and subtracted from the 16S rRNA distance matrix [35]. Briefly, for aforementioned 122 reference genomes, 16S rRNA sequences were obtained from Ribosomal Database (http://rdp.cme.msu.edu/seqcart/view.spr) using NCBI genome accession numbers [70]. These obtained sequences were aligned using ClustalW and phylogenetic distance matrix was computed. The 16S rRNA distance matrices obtained were then compared as above (mirrortree) with distance matrices of all the *E. coli* proteins. The highest correlation coefficient value between 16S rRNA distance matrix and *E. coli* protein was obtained and hereafter referred as “scaling factor” [35]. The scaling factor obtained was used to re-scale the protein distance matrices as well as 16S rRNA distance matrix by dividing each distance. The 16S rRNA distance matrix values were then subtracted from the corresponding protein distance matrices. These re-scaled distance matrices were then used to calculate PCC between protein pair as described above for mirrortree.

In GD-mirrortree method, we used a novel approach that is similar to Tol-mirrortree, however, the correction of protein distance matrices was done by subtracting the genome distances (GD) of the corresponding reference genomes from the distance values of the protein matrices. Genome distances for a pair of reference genomes were calculated using the following equation,


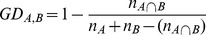


Where, n_A_ and n_B_ is the total number of proteins present in genomes A and B, respectively. *n*_A∩B_ is the number of orthologs shared by species A and B. The orthologs were obtained for each of the species using a bi-directional BLAST search against the remaining 121 reference genomes. The same procedure as above (Tol-mirrortree) was used to re-scale the protein matrices and genome distance matrix. However, the scaling factor was obtained by comparing genome distance matrix with protein matrices. The genome distance matrix values were then subtracted from protein distance matrices. This approach was referred as GD-mirrortree. Since the objective of this study was to understand the effect of reference genome selection on the performance of prediction methods, we made three reference genome sets using subsets of total 122 organisms of BAS set. We calculated interaction scores for gold standard protein pairs using their orthologs from each reference genome set by mirrortree, Tol-mirrortree and GD-mirrortree.

### Performance evaluation

Since the interaction scores were generated only for gold standard dataset protein pairs, we knew whether a particular pair was true positive, i.e., an interacting or true negative, i.e., a potentially non-interacting pair. These labels and corresponding interaction scores were then utilized to plot ROC and PR curves using ROCR package for R (http://www.r-project.org/) [71]. ROC curve visually represents the relative trade-offs between the FPR and the TPR [72]. A correct PPI prediction method would have a ROC curve above diagonal and its integral, AUC would be above 0.5. For 100% correct predictions, this curve is rectangular and AUC is equal to 1. PR curve visually represents the relative trade-offs between the precision and recall (or TPR). The TPR, FPR and precision values were calculated for a series of sorted interaction score thresholds of each prediction as below,


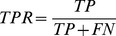



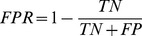



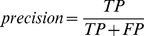


Where, TP and TN are the number of predicted true positives and true negatives for a particular confidence score threshold of PPI prediction method respectively. FP and FN are the number of predicted false positives and false negatives, respectively, for a particular confidence score threshold.

### Pathway Similarity

The KEGG database classifies proteins into various pathways which are associated with each protein in genome. Since a protein may belong to more than one pathway, Jaccard Coefficient (*JC*) of their KEGG pathway annotation was calculated as follows,


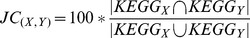


Where KEGG_x_ and KEGG_Y_ are the sets of specific pathways to which proteins X and Y belongs. The coefficient represented the degree by which two proteins share pathways [30].

## Supporting Information

Figure S1 **ROC curves for different reference genome sets for protein-protein interactions prediction methods on LQG dataset.** (**A**) ROC curves for six reference genome sets using Phylogenetic Profiling Methods. The solid lines depict the phylogenetic profile constructed using normalized bit scores (SPPM) whereas the dotted lines depict the binary phylogenetic profile (BPPM). The colors of the lines correspond to the six reference genome sets (ALL, BAAC, BAS, BAC, GAMMA and BANR) for which performance was evaluated. As evident in the figure, SPPM gives superior performance compared to BPPM for all reference genome sets. The ROC curves clearly show that the reference genome selection has profound influence on the performance of BPPM compared to that of SPPM. (**B**) ROC curves for six reference genome sets using Minimum Distance Method. The colors of the lines correspond to the six reference genome sets (ALL, BAAC, BAS, BAC, GAMMA and BANR) for which performance was evaluated. ROC plot shows that the method is broadly robust against choice of reference genome sets. All reference sets performed equally well except BANR which was slightly inferior. (**C**) ROC curves for four reference genome sets using Mirrortree based methods. We have used here two variants of the mirrortree methods i.e. the Tol-mirrortree and GD-mirrortree. The Tol-mirrortree (represented by dotted lines in the plot) uses 16S rRNA distance between two genomes as a factor to correct the phylogenetic distance whereas the GD-mirrortree (represented by solid lines in the plot) uses a genomic distance parameter reflecting the shared orthologs between two genomes to correct the corresponding phylogenetic distance (See methods for detail). The colors of the lines correspond to four reference genome sets (BAS, BAC, GAMMA and BANR) for which performance was evaluated. The plot clearly shows that the GD-mirrortree method performed slightly better compared to Tol-mirrortree method for these four reference genome sets.
(TIF)

Figure S2 **Precision-Recall (PR) plots for different reference genome sets for protein-protein interactions prediction methods.** (**A**) PR curves for six reference genome sets using Phylogenetic Profiling Methods on HQG dataset. The solid lines depict the phylogenetic profile constructed using normalized bit scores (SPPM) whereas the dotted lines depict the binary phylogenetic profile (BPPM). The colors of the lines correspond to the six reference genome sets (ALL, BAAC, BAS, BAC, GAMMA and BANR) for which performance was evaluated. As evident in the figure, SPPM gives superior performance compared to BPPM for all reference genome sets. The PR curves clearly show that the reference genome selection has profound influence on the performance of BPPM compared to that of SPPM. (**B**) PR curves for six reference genome sets using Gene Cluster Method on KEGG dataset. The colors of the lines correspond to the six reference genome sets (ALL, BAAC, BAS, BAC, GAMMA and BANR) for which performance was evaluated. The reference genome set GAMMA outperforms others however the PR curves diverge at higher recall values. (**C**) PR curves for six reference genome sets using Minimum Distance Method on HQG dataset. The colors of the lines correspond to the six reference genome sets (ALL, BAAC, BAS, BAC, GAMMA and BANR) for which performance was evaluated. PR plot shows that the method is robust against choice of reference genome sets. All reference sets performed equally well. (**D**) PR curves for four reference genome sets using Mirrortree based methods on HQG dataset. We have used here two variants of the mirrortree methods i.e. the Tol-mirrortree and GD-mirrortree. The Tol-mirrortree (represented by dotted lines in the plot) uses 16S rRNA distance between two genomes as a factor to correct the phylogenetic distance whereas the GD-mirrortree (represented by solid lines in the plot) uses a genomic distance parameter reflecting the shared orthologs between two genomes to correct the corresponding phylogenetic distance (See methods for detail). The colors of the lines correspond to the four reference genome sets (BAS, BAC, GAMMA and BANR) for which performance was evaluated. The plot clearly shows that the GD-mirrortree method is superior to Tol-mirrortree method for these four reference genome sets. For GD-mirrortree method BAS and BAC perform better than GAMMA and BANR.
(TIFF)

Table S1 Performance summary for four computational methods using different reference genome sets on LQG dataset.
(PDF)

Table S2 Performance summary for four computational methods using different reference genome sets on DIP protein-protein interactions.
(PDF)

Table S3 Performance summary for four computational methods using different reference genome sets on EcoCyc co-complex protein-protein interactions.
(PDF)

Table S4 Performance summary for Gene Cluster Method (GCM) at various Intergenic Distance Cutoffs (IDC) on KEGG pathway associations as benchmark for six reference genome sets.
(PDF)
