# Supplementary figures and images for: Effect of Reference Genome Selection on the Performance of Computational Methods for Genome-Wide Protein-Protein Interaction Prediction
Source: PLoS One. 2012 Jul 26;7(7):e42057. doi: 10.1371/journal.pone.0042057 (PMC3406042; doi:10.1371/journal.pone.0042057)

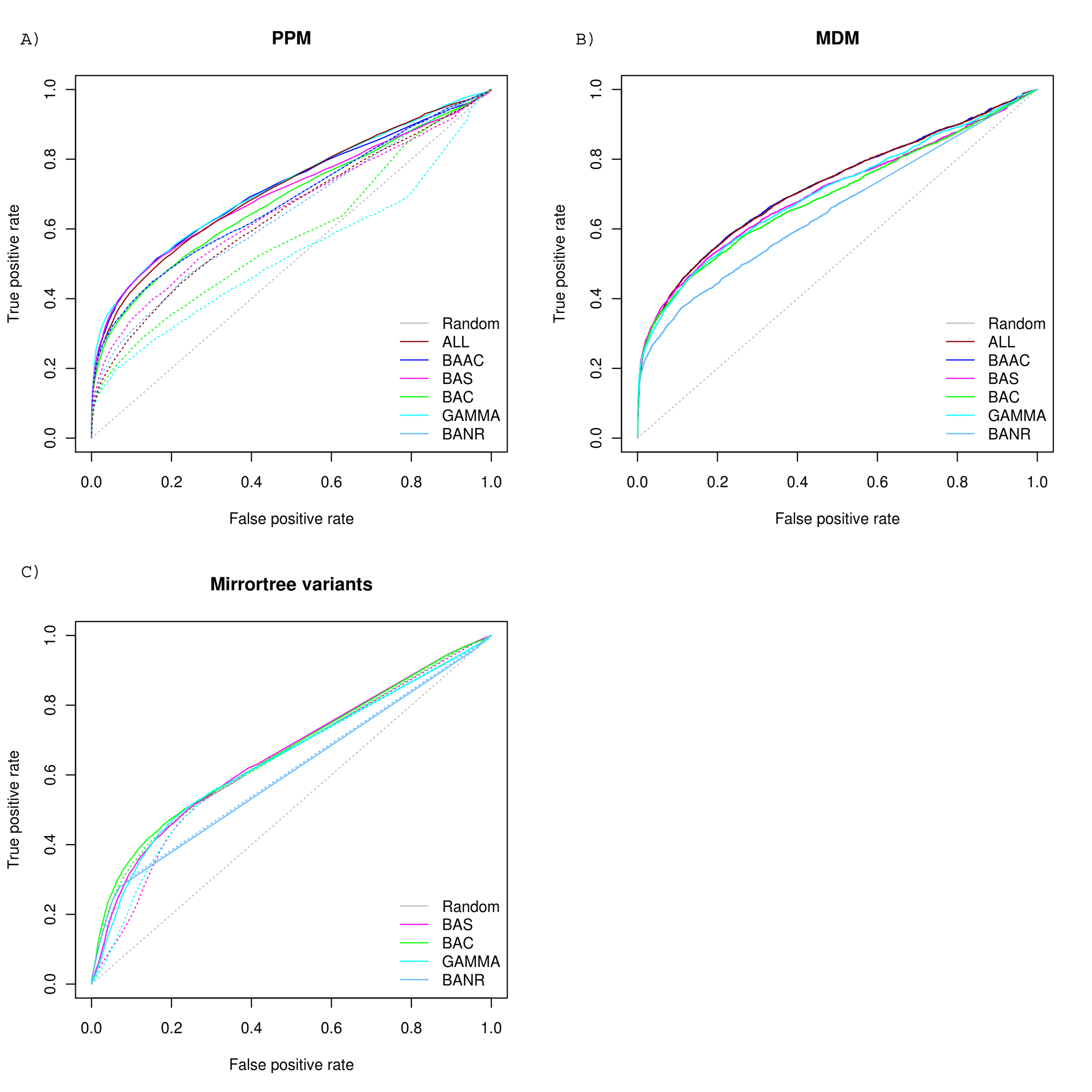

Supplement: Figure S1 — ROC curves for different reference genome sets for protein-protein interactions prediction methods on LQG dataset. (A) ROC curves for six reference genome sets using Phylogenetic Profiling Methods. The solid lines depict the phylogenetic profile constructed using normalized bit scores (SPPM) whereas the dotted lines depict the binary phylogenetic profile (BPPM). The colors of the lines correspond to the six reference genome sets (ALL, BAAC, BAS, BAC, GAMMA and BANR) for which performance was evaluated. As evident in the figure, SPPM gives superior performance compared to BPPM for all reference genome sets. The ROC curves clearly show that the reference genome selection has profound influence on the performance of BPPM compared to that of SPPM. (B) ROC curves for six reference genome sets using Minimum Distance Method. The colors of the lines correspond to the six reference genome sets (ALL, BAAC, BAS, BAC, GAMMA and BANR) for which performance was evaluated. ROC plot shows that the method is broadly robust against choice of reference genome sets. All reference sets performed equally well except BANR which was slightly inferior. (C) ROC curves for four reference genome sets using Mirrortree based methods. We have used here two variants of the mirrortree methods i.e. the Tol-mirrortree and GD-mirrortree. The Tol-mirrortree (represented by dotted lines in the plot) uses 16S rRNA distance between two genomes as a factor to correct the phylogenetic distance whereas the GD-mirrortree (represented by solid lines in the plot) uses a genomic distance parameter reflecting the shared orthologs between two genomes to correct the corresponding phylogenetic distance (See methods for detail). The colors of the lines correspond to four reference genome sets (BAS, BAC, GAMMA and BANR) for which performance was evaluated. The plot clearly shows that the GD-mirrortree method performed slightly better compared to Tol-mirrortree method for these four reference genome sets. (TIF) [file pone.0042057.s001.tif]

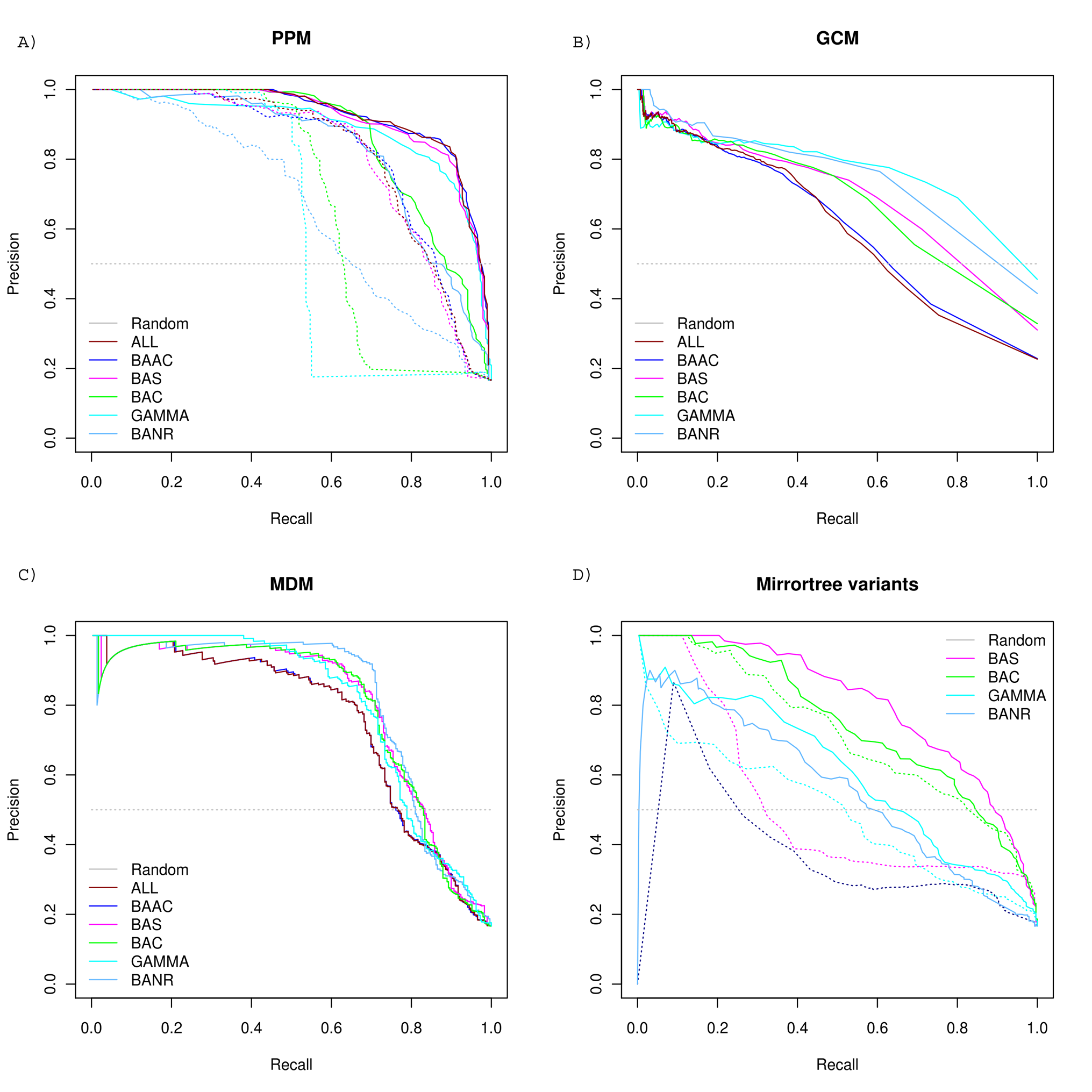

Supplement: Figure S2 — Precision-Recall (PR) plots for different reference genome sets for protein-protein interactions prediction methods. (A) PR curves for six reference genome sets using Phylogenetic Profiling Methods on HQG dataset. The solid lines depict the phylogenetic profile constructed using normalized bit scores (SPPM) whereas the dotted lines depict the binary phylogenetic profile (BPPM). The colors of the lines correspond to the six reference genome sets (ALL, BAAC, BAS, BAC, GAMMA and BANR) for which performance was evaluated. As evident in the figure, SPPM gives superior performance compared to BPPM for all reference genome sets. The PR curves clearly show that the reference genome selection has profound influence on the performance of BPPM compared to that of SPPM. (B) PR curves for six reference genome sets using Gene Cluster Method on KEGG dataset. The colors of the lines correspond to the six reference genome sets (ALL, BAAC, BAS, BAC, GAMMA and BANR) for which performance was evaluated. The reference genome set GAMMA outperforms others however the PR curves diverge at higher recall values. (C) PR curves for six reference genome sets using Minimum Distance Method on HQG dataset. The colors of the lines correspond to the six reference genome sets (ALL, BAAC, BAS, BAC, GAMMA and BANR) for which performance was evaluated. PR plot shows that the method is robust against choice of reference genome sets. All reference sets performed equally well. (D) PR curves for four reference genome sets using Mirrortree based methods on HQG dataset. We have used here two variants of the mirrortree methods i.e. the Tol-mirrortree and GD-mirrortree. The Tol-mirrortree (represented by dotted lines in the plot) uses 16S rRNA distance between two genomes as a factor to correct the phylogenetic distance whereas the GD-mirrortree (represented by solid lines in the plot) uses a genomic distance parameter reflecting the shared orthologs between two genomes to correct the corresponding phylogenetic [file pone.0042057.s002.tif]
