# Supplementary material for: Effect of Reference Genome Selection on the Performance of Computational Methods for Genome-Wide Protein-Protein Interaction Prediction
Source: PLoS One. 2012 Jul 26;7(7):e42057. doi: 10.1371/journal.pone.0042057 (PMC3406042; doi:10.1371/journal.pone.0042057)
Supplement: Table S2 — Performance summary for four computational methods using different reference genome sets on DIP protein-protein interactions. (PDF) [file pone.0042057.s004.pdf]

Table S2. Performance summary for four computational methods using different reference genome sets on DIP protein-protein interactions

Method	Variant	ALL	BAAC	BAS	BAC	GAMMA	BANR
PPM	BPPM	0.70	0.73	0.74	0.61	0.48	0.72
	SPPM	0.86	0.88	0.87	0.85	0.82	0.79
GCM	GCM	0.90	0.89	0.92	0.91	0.94	0.82
MDM	MDM	0.80	0.80	0.81	0.81	0.81	0.74
Mirrortree	Mirrortree	NA	NA	0.75	0.77	0.76	0.69
	Tol-mirrortree	NA	NA	0.74	0.77	0.77	0.67
	GD-mirrortree	NA	NA	0.78	0.78	0.79	0.65

Notes: The performance summary of protein-protein prediction methods measured as Area Under the ROC Curve (AUC). BPPM stands for Binary Phylogenetic Profile Method; SPPM stands for Sequence Similarity (Bits scores) based Phylogenetic Profiling Method; GCM is Gene Cluster Method, MDM is gene neighborhood based Minimum Distance Method; GD is genome distance; NA stands for sets that are not analyzed for corresponding methods. ALL, BAAC, BAS, BAC, GAMMA and BANR are reference genome sets whose compositions is given in Table 1.
