# Supplementary material for: Effect of Reference Genome Selection on the Performance of Computational Methods for Genome-Wide Protein-Protein Interaction Prediction
Source: PLoS One. 2012 Jul 26;7(7):e42057. doi: 10.1371/journal.pone.0042057 (PMC3406042; doi:10.1371/journal.pone.0042057)
Supplement: Table S4 — Performance summary for Gene Cluster Method (GCM) at various Intergenic Distance Cutoffs (IDC) on KEGG pathway associations as benchmark for six reference genome sets. (PDF) [file pone.0042057.s006.pdf]

Table S4. Performance summary for Gene Cluster Method (GCM) at various Intergenic Distance Cutoffs (IDC) on KEGG pathway associations as benchmark for six reference genome sets

IDC	ALL	BAAC	BAS	BAC	GAMMA	BANR
100	0.76	0.76	0.78	0.76	0.8	0.75
200	0.75	0.75	0.77	0.75	0.79	0.75
300	0.73	0.74	0.76	0.75	0.8	0.76
400	0.72	0.73	0.75	0.74	0.8	0.76
500	0.71	0.72	0.74	0.73	0.8	0.76

Notes: The performance of GCM is higher for GAMMA and BAC reference genome sets. IDC of 100 nucleotide bases is seems to be optimal distance between adjacent genes to define gene clusters in the reference genomes. The performance summary of GCM measured as Area Under the ROC Curve (AUC). ALL, BAAC, BAS, BAC, GAMMA and BANR are reference genome sets whose compositions is given in Table 1. The proteins that share at least one KEGG served as positives examples otherwise negative examples.
